# flippant–An R package for the automated analysis of fluorescence-based scramblase assays

**DOI:** 10.1186/s12859-017-1542-y

**Published:** 2017-03-03

**Authors:** Richard J. Cotton, Birgit Ploier, Michael A. Goren, Anant K. Menon, Johannes Graumann

**Affiliations:** 1 Research Division, Weill Cornell Medicine–Qatar, P.O.Box 24144, Doha, State of Qatar; 2 Department of Biochemistry, Weill Cornell Medical College, New York, NY 10065 USA; 3 Current Address: Scientific Service Group Biomolecular Mass Spectrometry, Max Planck Institute for Heart and Lung Research, W.G. Kerckhoff Institute, Ludwigstr. 43, D-61231 Bad Nauheim, Germany

**Keywords:** Scramblase, Dithionite scramblase assay, R

## Abstract

**Background:**

The lipid scrambling activity of protein extracts and purified scramblases is typically measured using a fluorescence-based assay. While the assay has yielded insight into the scramblase activity in crude membrane preparations, functional validation of candidate scramblases, stoichiometry of scramblase complexes as well as ATP-dependence of flippases, data analysis in its context has remained a task involving many manual steps.

**Results:**

With the extension package “flippant” to R, a free software environment for statistical computing and graphics, we introduce an integrated solution for the analysis and publication-grade graphical presentation of dithionite scramblase assays and demonstrate its utility in revisiting an originally manual analysis from the publication record, closely reproducing the reported results.

**Conclusions:**

“flippant” allows for quick, reproducible data analysis of scramblase activity assays and provides a platform for review, dissemination and extension of the strategies it employs.

**Electronic supplementary material:**

The online version of this article (doi:10.1186/s12859-017-1542-y) contains supplementary material, which is available to authorized users.

## Background

Scramblases are proteins mediating the bidirectional, mass-action driven equilibration of lipids between the leaflets of lipid bilayers that constitute biological membranes [1–3]. Independent of energy equivalents such as ATP they facilitate this “flipping” by providing a mechanism by which polar lipid head groups can transition through the hydrophobic environment of membranes, thus reducing the massive energetic cost this process would carry otherwise [4]. The underlying molecular mechanisms remain largely enigmatic [5]. Proteins supporting flipping processes as central as the equilibration of the ubiquitous phosphoglycerolipids across the membrane of the endoplasmic reticulum, where they are synthesized in the cytoplasmic monolayer, have yet to be identified [6]. Nonetheless, there has been much recent progress in scramblase identification and characterization. Proteins shown to have scramblase activity include FtsW [7], opsin/rhodopsin [8, 9], TMEM16 [10], *β*2 -adrenergic and adenosine A2A receptors [9]. Extrapolating from the latter findings and the opsin/rhodopsin case, one may speculate that lipid scrambling may, in fact, be an intrinsic property of G-protein coupled receptors in general [9].

To interrogate scramblases as a functional class of proteins, a biochemical assay was developed suited to characterize scramblase activity of purified proteins and protein extracts based on the reconstitution of scramblases or candidate proteins into preformed synthetic liposomes that contain trace amounts of nitrobenzoxadiazole (NBD) labeled fluorescent lipids [11]. Upon addition of membrane-impermeable reducing agents such as dithionite to these liposomes, fluorophores in the external leaflet are reduced and fluorescence in scramblase-free liposomes accordingly quickly drops to approximately 50% of the initial value. However, for proteoliposomes reconstituted with proteins conferring a scramblase activity, fluorescence disappears almost entirely, as fast scramblase-mediated equilibration between outer (exposed) and inner (protected) leaflet of the bilayer renders all fluorescent lipids accessible to reduction. By titration of the protein amount reconstituted into the liposome membranes it is thus possible to characterize scramblases and probe their activity. Beyond the identification of scramblase activities in crude membrane preparations [11, 12] and functional validation of candidate scramblases [8–10, 13], the assay has also been used to characterize the stoichiometry of scramblase homo-multimers reconstituting into the proteoliposomes [14] and probe the ATP-dependence of flippases, molecules that under energy-consumption flip lipids against a concentration gradient between bilayer leaflets [15].

Starting with the identification of baseline and post-reduction fluorescence from fluorimeter-generated spectra, via the calculation of the underlying statistics and through publication-grade representation of the results, data analysis in the context of this assay has until now involved an extensive series of manual steps, at most supported by spreadsheet calculation facilities [8, 9, 14]. Based on the R free software environment for statistical computing and graphics [16] and prominently drawing on the power and elegance of the ggplot2 plotting system [17], we here provide “flippant”, a package that comprehensively, fast and reproducibly covers all data analysis and graphing needs from raw fluorescence spectra produced by scramblase assays. A simple parametrizing spreadsheet is the sole required user input.

## Implementation

“flippant” has been implemented in R [16] and is available in The Comprehensive R Archive Network (CRAN). It is thus install- and loadable as follows from within a functional R environment:
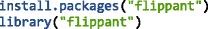


The package depends on the following packaged extensions to R base functionality, which are automatically satisfied on installation: ggplot2, assertive.files, assertive.numbers, assertive.properties, assertive.strings, assertive.types, data.table, magrittr, minpack.lm, plyr, RcppRoll, stringi, utils, withr and wmtsa.

## Input data and data processing

To analyze raw data stemming from dithionite scramblase assays, “flippant” requires for each data point information on the path to the fluorimeter-generated spectrum, the amount of protein reconstituted into proteoliposomes, the assay volume before and after addition of dithionite (to calculate a volume correction factor), the amount of lipids present (to calculate a protein to phospholipid ratio or PPR) etc. The user supplies this information in a spreadsheet (see below for supporting functionality).

Spectral input data is read by “flippant” using the file paths provided in the spreadsheet. Raw spectra produced by QuantaMaster fluorimeter instruments (Photon Technology International, Inc., Edison, New Jersey) running software versions FelixGX v4.1 and Felix32 v1.20, respectively, as well as a simple generic tab-delimited spectrum representing file format with time (in seconds) and fluorescence intensity representing columns are currently supported. The latter is trivially assembled from any fluorescence data using a spreadsheet program. File type determination is handled algorithmically.

Based on [8, 9] as well as [14] and largely keeping with nomenclature used therein, data are processed as follows. Input is format checked and defaults are injected for facultative parameters/columns as appropriate. Fluorescence spectra are parsed. This includes algorithmic determination of when dithionite was added to the sample using peak detection by continuous wavelet transformation as implemented in the wmtsa package [18, 19]. Acquisition time is aligned for all spectra in the data set such that the zero time point henceforth corresponds to the time of addition. Pre-dithionite-addition baseline fluorescence *F*_*baseline*_ is determined for each spectrum by averaging (median) over the ten values preceding dithionite addition. Post-dithionite-addition minimum fluorescence *F*_*min*_ is analogously calculated from the last ten data points before (and including) a default 400 s or the time point of measurement supplied by the user in the parameter spreadsheet. *F*_*min*_ is volume-corrected based on supplied reaction volumes with and without dithionite and for each spectrum/data point a fluorescence reduction is calculated as follows:

$$ {y}_{raw}=1-\frac{F_{min}}{F_{baseline}} $$

A relative fluorescence reduction *y* is calculated scaled to a (required) liposomes-only/no-protein control and the probability *P*_≥ 1_ for a liposome to contain at least one scramblase molecules is calculated using

$$ {P}_{\ge 1}=\frac{\left( y-{y}_0\right)}{\left({y}_{max}-{y}_0\right)} $$

where *y* is the relative fluorescence reduction for the proteoliposome measurement and *y*_0_ for a measurement of protein-free liposomes. *y*_*max*_ refers to either the maximal *y* in the experiment or is derived from a monoexponential fit to the data (default) as a precaution for the case where the protein/phospholipid titration did not reach the plateau of the saturation curve. The behavior is adjustable using the scale_to parameter of the corresponding functions (see below). Next a monoexponential curve is fitted to the relationship of *P*_≥ 1_ to the protein to phospholipid ratio (PPR; (mg/mmol)), providing the scramblase-characterizing fit factor *α*. By default and following [14], PPR is scaled by a factor of 0.65 to account for a fraction of the vesicle pool used that is refractory to scramblase reconstitution. This behavior can be modified using the ppr_scale_factor parameter and avoided altogether setting it to NULL.

## Main features

### Template generation for data input

Supporting the user in providing the input data needed, the following function generates a spreadsheet program-compatible tab-delimited plain text template, including column names, commentary, expected data type and the default used when omitted.
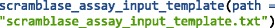


The application agnostic tab-delimited format must be maintained for the completed table, which is subjected to extensive input checks when read by “flippant”.

### Graphic representation of spectral traces

In a first, graphical data analytic step of a scramblase assay-derived data set, fluorescent spectra or traces may be plotted in publication-appropriate quality with acquisition time on the x- and fluorescence intensity on the y-axis using
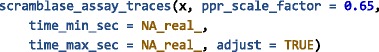


and handing the function the path to the user assembled spreadsheet of input data (x). The adjust argument to the function call serves to selectively inactivate wmtsa-provided (see above) alignment of multiple spectra to the time of dithionite addition, while the ppr_scale_factor parameter is used to scale PPR (see above) and is needed here as the traces are colored by that measure. Acquisition time coverage of the plot may be tuned by user-provided minimal (time_min_sec) and maximal (time_max_sec) time points to be included (in seconds).

### Scramblase assay analysis

Plotting protein to phospholipid ratio (PPR) against the likelihood for a given proteoliposome to contain one or more scramblases, the PPR plot is at the heart of the analysis of dithionite scramblase assays, allowing for the visual comparison of specific scramblase activity between interrogated experimental series, which may, for example, be representative of protein preparations including mutant protein forms or crude extracts with or without depletion of a candidate scramblase.
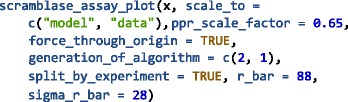


allows for the complete generation of the PPR plot in publication quality from the user assembled spreadsheet of input data (x). The behavior of the function may be adjusted to use scramblase-mediated fluorescence reduction normalized to either the maximum measurement in the series (scale_to = "data") or the plateau of an exponential fit to it (scale_to = "model"; default; for reasoning see above). ppr_scale_factor allows for the modification of scaling to account for a pool of vesicles that is scramblase-inaccessible (see above). The formula to which the data is fitted has evolved over time and “flippant” supports both the original ([8, 9]; generation_of_algorithm = 1), as well as the more recent iteration ([14]; default; generation_of_algorithm = 2), which takes into account the size distribution of the liposome population used and thus also requires parameters for liposome radius and its standard deviation (r_bar and sigma_r_bar, respectively; in nm, both). The split_by_experiment argument is used to indicate whether independent experiments are integrated into a single analysis (split_by_experiment = FALSE) or treated separately (split_by_experiment = TRUE; see below for example usage). The force_through_origin parameter may be used to employ a fit with more degrees of freedom. While the non-default force_through_origin = FALSE allows for a better fit to some experimental data, its mechanistic implications are unclear.

Numeric scramblase assay results such as *α* may be generated in tabular form using
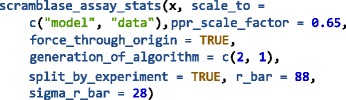


with the same modifying arguments to the function as for scramblase_assay_plot.

## Results and discussion

*Retinitis pigmentosa* is a degenerative disease of the retina, the majority of cases of which is linked to mutant forms of the G-protein coupled receptor rhodopsin [20]. Motivated by the recent discovery that opsin/rhodopsin has scramblase activity [8, 9], we hypothesized that a class of enigmatic rhodopsin mutations, which are known to cause *retinitis pigmentosa* yet do not display the commonly associated molecular phenotypes of impaired binding of retinal, folding and/or trafficking or transducin activation, may in fact be impacted in their scramblase activity. This hypothesis was disproved. However, the same set of experiments pointed at a defect in rhodopsin dimerization prior to insertion into liposomes, generating the new hypothesis that the mutations may cause disease by interfering with the highly ordered quarternary structure of rhodopsin in the retina [14].

“flippant’s” capabilities are demonstrated here by reanalyzing a subset of the data in [14]. The first step is to extract the data files, stored in a ZIP archive within the package.
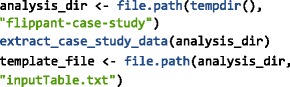


**Fig. 1:**
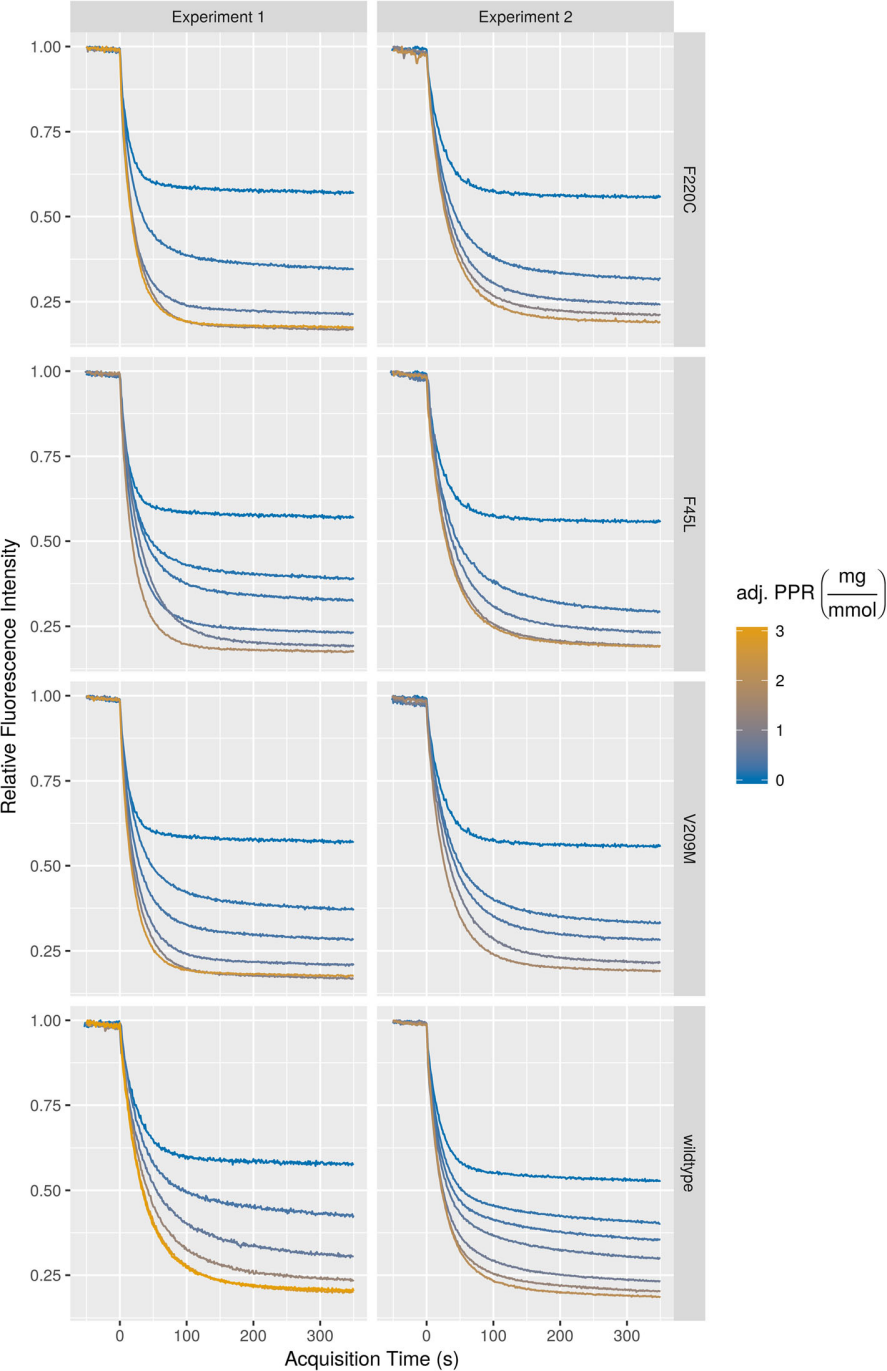
flippant-based plotting of spectral traces underlying a subset of the data in Figure 4C–F from [14]. See text for command and details


To begin the analysis it is useful to plot the spectral traces of each experimental series (Fig. 1). Note that algorithmic alignment to the time of dithionite addition is employed (default) as well as custom trimming of the time axis to account for divergent acquisition times between experiments. PPR scaling is used with the default factor of 0.65.

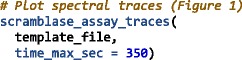


Scramblase activity analysis as described above is performed on the data set with the following calls.
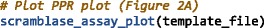


**Fig. 2:**
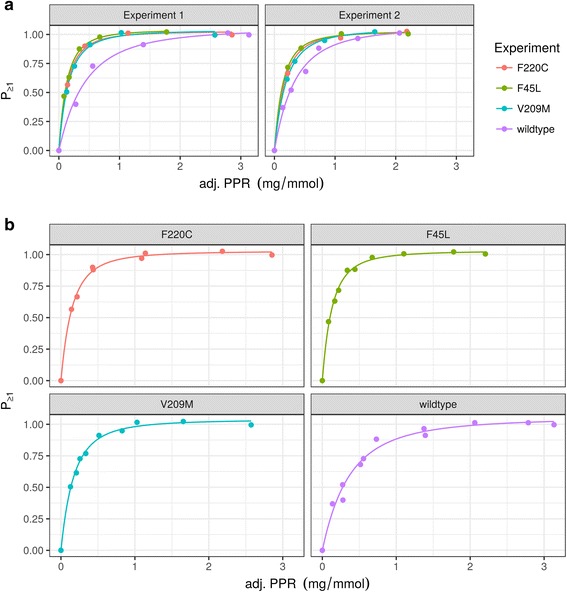
flippant-based reanalysis of a subset of the data in Fig. 4C–F from [14]. (**a**) and (**b**) show protein to phospholipid ration (PPR) plots for the data using a x-axis trimmed to a maximum value of 3 (see text for details). In (**a**) data is separately plotted for independent experiments, while (**b**) combines all data points, aiming for reliability of fit. ‘adj. PPR’ indicates that the measure has been scaled to account for a vesicle pool refractory to flippase reconstitution

The call results in the protein to phospholipid ratio (PPR) plot shown in Fig. 2a.

Figure 2a emphasizes reproducibility between independent experiments (“Experiment 1” and “Experiment 2”) on the four probed rhodopsins. Forgoing reproducibility analysis in favor of a potentially more reliable result and following more closely the analysis by [14], a single fit may be performed for the combined data from all experiments as follows, resulting in Fig. 2b:
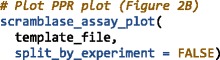


The graphical representation of the fit constant *α* in the PPR plots is difficult to visually evaluate and easy access to the numeric values desirable. Recapitulation of the calculations underlying PPR plot generation for Fig. 2b by scramblase_assay_plot in a call to scramblase_assay_stats, this is achieved and produces tabular output represented in Table 1.

**Table 1:** Tabular output of flippant-based reanalysis of a subset of the data in Fig. 4C–F from [14]. An plain text-version of this output is produced by calling scramblase_assay_stats

Experimental series	Fit constant (× 10^4^)
F220C	14.92
F45L	16.98
V209M	13.06
Wildtype	6.17


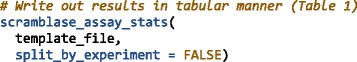


The resulting values for the fit constant correspond with 14.92, 16.98, 13.06 and 6.17 for F220C, F45L, V209M and wildtype, respectively, well to the prior manual analysis by [14], where *α* × 10^4^ ≈ 16 was reported for mutant and *α* × 10^4^ ≈ 7 for wild type rhodopsin. Based on the manual analysis of the data we conclude in [14] that the mutant rhodopsins investigated are reconstituted into proteoliposomes in monomeric form, contrary to the wild type, which is reconstituting as a homodimer. “flippant” analysis of the data shows identical trends and close matches of the results, supporting identical conclusions.

## Conclusions

With “flippant” we present an integrated solution for data analysis in the context of dithionite scramblase assays. Requiring only basic familiarity with R as an environment for statistical computing and graphics [16], scientists can quickly analyze such data and arrive at publication-grade graphics that offer extensive facilities for individual optimization and adaptation [17]. On a laptop system with 8 cores and 8 GB RAM (no SSD), running Linux and R version 3.3.2, the statements generating equivalents to the plots and calculations presented here take a mere 24 s (median; *MAD* = 0.18 *s*; *n* = 100).

Results from “flippant”-driven analysis track prior manual analysis well. Deviations observed may stem from algorithmic differences such as the fitting routine used (“flippant” uses the the Marquardt nonlinear least squares algorithm as implemented by [21]) and general handling of significant digits and rounding. Another potential source of minor divergence is the algorithmic versus manual determination of the time point at which reductant is added in an experiment, potentially resulting in slightly differing determination of relative fluorescence reduction.

The choice of R for the implementation is not only motivated by the extensive tool kit for statistic methodology and exquisite plotting environment it provides, but also by the fact that it is a scripting language, rendering the implementation unequivocally open to scrutiny and adaptation or extension by users.

“flippant”, in conclusion, provides means for quick, reproducible data analysis in the context of scramblase activity analysis and a platform for review, dissemination and extension of the strategies it employs. As BSA-back extraction, a different assay for lipid translocation across liposome bilayers [22, 23], can utilize an essentially identical data analysis work flow, “flippant” is expected to facilitate analysis of such experiments as well.

## Additional file

Additional file 1:
R source package of flippant in the version current at acceptance for publication. (GZ 191 kb)

## Abbreviations

ATP: Adenosine triphosphate
BSA: Bovine serum albumin
CRAN: The Comprehensive R Archive Network
MAD: Median absolute deviation
NBD: Nitrobenzoxadiazole
PPR: Protein to phospholipid ratio
RAM: Random-access memory
SSD: Solid state disk
